# Scientific Items

**Published:** 1885-09-20

**Authors:** 


### SCIENTIFIC ITEMS.

**A New Swedish Invention.**—The Swedish civil engineer, A. F. Westerlund, of Stockholm, has lately obtained letters patent of the United States for a very useful invention within the chemical and technical branches of science. It consists in the production of an almost incombustible coal, which stands between the graphite and the diamond, and is consequently named diamond coal. Its production is very simple and inexpensive, and the invention so important for both hygienic and technical purposes that it can almost be looked upon as the Columbian egg. This invention can be divided into two branches, one for hygienic and the other for technical purposes, which latter have not yet been fully compiled. The hygienic part is based principally upon the production of a coal in felt form, which through its antiseptic properties has created quite a sensation within the medical fraternity, both here and abroad. It is known under the name of carbon wadding. The highest testimonials of the first military surgeons of Sweden have been gladly given to the carbon wadding, and it has been introduced into the principal hospitals in London. It can be made from any vegetable substance, such as moss, hay, straw, cotton, paper, cork, wood shavings, etc.—*Scientific American.*

**Climatology of the Puget Sound Country.**—The inland waters of Washington Territory, Puget Sound, and its tributaries are frequently called the Mediterranean of the Pacific Coast, and justly so, for they are equally exempt from equinoctial storms. Since the Weather Bureau was established on this coast, the highest wind has reached a speed of only about forty miles an hour. During an observation of thirty-one years, the lowest temperature ever recorded was 10° above zero. The highest for the same length of time was below 90°. But in the interior and near the mountains, even but a little above the sea level, there are greater extremes of heat and cold.

On this northwestern coast there are no extremes of cold in winter or of heat in summer.—*Scientific American.*

**A New Use for Toads.**—The latest and most ingenious way of getting rid of roaches and water bugs we have heard of, is related of a citizen of Schenectady whose kitchen was infested with them.

A servant, hearing that toads were an antidote, caught three ordinary hop toads, and put them in the kitchen. Not a roach or water bug, it is stated, can now be found in the house. The toads have become domesticated, never wander about the house, and are so cleanly and inoffensive that there is no objection to their presence.

Another use for toads is to employ them for insect destroyers in the garden. They are determined enemies of all kinds of snails and slugs, which it is well known can in a single night destroy a vast quantity of lettuce, carrots, asparagus, etc. Toads are also kept in fineyards, where they devour during the night millions of insects that escapé the pursuit of nocturnal birds, and might commit incalculable havoc on the buds and young shoots of the vine. In Paris toads are an article of merchandise. They are kept in tubs, and sold at the rate of two francs a eozen.—*S. American.*

**Animal Eccentricities.**—If a man begins to save against his old age, we call it thrift, and praise him as a small capitalist who s giving hostages to fortune; but if a dog accumulates a store of bones or food, we look upon him as indulging in dangerous caprices, which may end in the necessity of putting a bullet through his head. There may be exceptions here and there. Sometimes you will find an old lady who will protect eccentricity in a parrot, a magpie, or a jackdaw, as a bird that has a right to a certain freedom of movement in return for its entertaining attempts at conversation. But, on the whole, there is no sterner standard of conventionality than that which we enforce on our domestic animals. Pet dogs become perfect bigots in favor of the usual, and persecute any attempt to deviate from it on the part even of a more powerful and less favored colleague, as the Inquisition persecuted heresy, or as the court of Russia Nihilism. There is nothing equal to the indignation of an in-doors dog at any invasion of the privacy of the drawing-room by an out-doors dog, and nothing more melancholy than the servile apologies which the big dog will make to the little one, for even proposing to break through the animan etiquette of the house.—*Popular Science Mothly.*

**A Surgical Instrument** has been patented by Mr. John S. Poynor, of Walnut Springs, Texas. In a head rod a series of springs is held with their inner ends to turn in such a way as to make an improved device for extracting foreign bodies from different parts of the body, and one which can be used as a probe.—*Scientific American.*

**A Life. Saving Apparatus** has been patented by Mr. Olney Arnold, of Pawtucket, R. I. Combined with a kite is a small boat, carrying a spool or bobbin with a life line, on one end of which is made fast to the shore, the boat being so made as to adapt it to increased or diminished resistance in the water, and so it can be guided out of alignment with exact direction of the wind.—*Scientifc American.*

**A Slicer** has been patented by Mr. Daniel J. Gilchrist, of Newark, N. J. Combined with a slide carrying a blade is a board held to the slide by links and an angle lever, a rod being connected with the angle lever by means of which the board can be moved a greater or less distance from the edge of the blade, and the device readily adjusted to cut slices of any desired thickness.

**A Machine** for straightening match splits has been patented by Mr. William H. Wyman, of Oshkosh, Wis.

